# Chromosome painting among Proboscidea, Hyracoidea and Sirenia: support for Paenungulata (Afrotheria, Mammalia) but not Tethytheria

**DOI:** 10.1098/rspb.2007.0088

**Published:** 2007-03-20

**Authors:** A.T Pardini, P.C.M O'Brien, B Fu, R.K Bonde, F.F.B Elder, M.A Ferguson-Smith, F Yang, T.J Robinson

**Affiliations:** 1 Evolutionary Genomics Group, Department of Botany and Zoology, University of StellenboschPrivate Bag X1, Matieland, 7602 Stellenbosch, South Africa; 2 Centre for Veterinary Science, University of CambridgeCambridge CB3 0ES, UK; 3 U.S. Geological Survey, Florida Integrated Science CentreGainesville, FL 32605-3574, USA; 4 Department of Pathology, Cytogenetics LaboratoryUT Southwestern Medical Centre, Dallas, TX 75235, USA

**Keywords:** zoo-fluorescence *in situ* hybridization, elephant, hyrax, manatee, Paenungulata, chromosomal evolution

## Abstract

Despite marked improvements in the interpretation of systematic relationships within Eutheria, particular nodes, including Paenungulata (Hyracoidea, Sirenia and Proboscidea), remain ambiguous. The combination of a rapid radiation, a deep divergence and an extensive morphological diversification has resulted in a limited phylogenetic signal confounding resolution within this clade both at the morphological and nucleotide levels. Cross-species chromosome painting was used to delineate regions of homology between *Loxodonta africana* (2*n*=56), *Procavia capensis* (2*n*=54), *Trichechus manatus latirostris* (2*n*=48) and an outgroup taxon, the aardvark (*Orycteropus afer*, 2*n*=20). Changes specific to each lineage were identified and although the presence of a minimum of 11 synapomorphies confirmed the monophyly of Paenungulata, no change characterizing intrapaenungulate relationships was evident. The reconstruction of an ancestral paenungulate karyotype and the estimation of rates of chromosomal evolution indicate a reduced rate of genomic repatterning following the paenungulate radiation. In comparison to data available for other mammalian taxa, the paenungulate rate of chromosomal evolution is slow to moderate. As a consequence, the absence of a chromosomal character uniting two paenungulates (at the level of resolution characterized in this study) may be due to a reduced rate of chromosomal change relative to the length of time separating successive divergence events.

## 1. Introduction

The enigmatic mammalian clade Paenungulata (Simpson 1945) is represented among extant mammals by the morphologically diverse orders of Hyracoidea (hyraxes), Proboscidea (elephants) and Sirenia (manatees, dugongs and sea cows). Despite extensive molecular and morphological analyses, this clade remains one of the unresolved (and controversial) nodes in mammalian systematic studies (Waddell *et al.* 2001; Gheerbrant *et al.* 2005). A central issue in the morphological debate concerns the phylogenetic position of Hyracoidea and consequently, the monophyly of Paenungulata. Specifically, this revolves around whether Simpson's (1945) unification of these three orders is more justifiable than the alternative hypothesis of a Sirenia–Proboscidea association (Tethytheria; McKenna 1975) with Hyracoidea more closely aligned with Perissodactyla (e.g. Fischer & Tassy 1993). The exclusion of Hyracoidea from Paenungulata on morphological grounds has significant implications for intrapaenungulate relationships as this indicates that the closer association of Proboscidea with Sirenia (Tethytheria) is more acceptable. In contrast, the monophyly of Paenungulata is well supported by large molecular datasets (Gheerbrant *et al.* 2005, p. 99) yet, unlike the morphological-based Tethytheria hypothesis, there is minimal consensus among different DNA markers regarding intrapaenungulate relationships (e.g. Murphy *et al.* 2001; Waddell *et al.* 2001; Amrine-Madsen *et al.* 2003). A pattern has emerged showing a disparity between mitochondrial and nuclear DNA with the largest concatenations of mitochondrial protein-coding segments favouring Tethytheria (Murata *et al.* 2003; Nikaido *et al.* 2003). While the results from different nuclear DNA markers vary, nuclear amino acid sequence comparisons tend to favour a Hyracoidea–Proboscidea association (Murphy *et al.* 2001; Waddell *et al.* 2001; Nishihara *et al.* 2005).

The difficulty in resolving the paenungulate polytomy is probably due to the rapid radiation of Paenungulata resulting in a limited time for synapomorphies to be established (Amrine & Springer 1999; Nishihara *et al.* 2005). This situation is exacerbated by the relatively deep divergence estimated at approximately 62 Myr ago (Springer *et al.* 2003), which increases the opportunity for homoplasy to reduce the likely already limited phylogenetic signal for reconstructing evolutionary relationships. A further consideration, pertinent to morphological analysis in particular, is the extensive diversification that has occurred within each of the three lineages. This is typified by highly specialized niche adaptations, an example of which is the complete adaptation to an aquatic habitat seen in Sirenia, leading to the masking of characters by autapomorphic changes and to morphological convergence confounding morphological datasets for Tethytheria (Robinson & Seiffert 2004 and references therein).

The lack of consensus between molecular studies and the problems associated with morphological characters in resolving these relationships indicates the difficulty of current markers for elucidating relationships within Paenungulata. The comparison of patterns of chromosomal change between taxa is proving to be a useful tool in understanding evolutionary relationship and has already been shown to be effective in delineating clade-specific cytogenetic signatures, including Afrotheria (Frönicke *et al.* 2003; Yang *et al.* 2003*a*; Robinson *et al.* 2004). These changes, which belong to a class of marker called rare genomic changes (RGCs; Rokas & Holland 2000), offer specific advantages. For example, they are large scale and infrequent in comparison with nucleotide changes in sequence data (Rokas & Holland 2000) and thus they reduce problems associated with homoplasy. As with morphological characters, chromosomal rearrangements can be scored on a simple presence–absence basis (Dobigny *et al.* 2004) and hence avoid a complex analytical approach required for phylogenetic analysis of sequence data (Waddell *et al.* 2001; Amrine-Madsen *et al.* 2003).

In the present study, we provide reciprocal cross-species chromosome painting data among representative taxa for each of the three paenungulate orders and for each of these to the outgroup taxon, the aardvark (*Orycteropus afer*). Through the delineation of chromosomal homologies among the four afrotherian taxa, as well as an indirect comparison with an additional outgroup (the human), we endeavoured to address the following aims: (i) to identify cytogenetic signatures that consolidate Paenungulata, (ii) to characterize synapomorphic changes to define intrapaenungulate relationships, and (iii) through the reconstruction of an ancestral paenungulate karyotype (APK), to provide insight into paenungulate chromosomal evolution.

## 2. Material and methods

### (a) Chromosome and standard karyotype preparation

Chromosome preparations were made from fibroblast cultures derived from a Florida manatee (male *Trichechus manatus latirostris*, TMA, from Florida, USA), an African elephant (male *Loxodonta africana*, LAF, from Namibia), a Cape rock hyrax (male *Procavia capensis*, PCA, South Africa) and an aardvark (male *Orycteropus afer*, OAF, South Africa). The numbering and organization of the respective G-banded karyotypes followed previous studies (elephant, Houck *et al.* 2001; manatee, Gray *et al.* 2002; aardvark, Yang *et al.* 2003*a*), the exception being *P. capensis*. Following a comparison between the hyrax karyotype presented here and that recently published by Froenicke (2006), intrachromosomal differences were found between several chromosomes (table S1 in electronic supplementary material), making some of the homology comparisons problematic. To avoid problems of misidentification, the karyotype presented here is from the South African specimen referred to above; autosomes were grouped on the basis of the centromere position (meta/submetacentric and acrocentric) and ordered by decreasing size.

### (b) Fluorescence *in situ* hybridization

#### (i) Flow sorting and generation of chromosome-specific paint probes

Preparations of flow-sorted chromosomes for the elephant have previously been described (Yang *et al.* 2003*a*). Chromosome-specific painting probes for the manatee and hyrax were generated from flow-sorted suspensions of Hoechst 33258- and chromomycin A3-stained chromosomes on the basis of size and AT : GC ratio and subsequently amplified by degenerate oligonucleotide-primed polymerase chain reaction (DOP-PCR; Telenius *et al.* 1992; Ferguson-Smith *et al.* 1998). The identities of the flow-sorted chromosomes were determined by hybridization to G-banded metaphase spreads of the donor species using fluorescence *in situ* hybridization (FISH).

#### (ii) Cross-species chromosome painting

Chromosome painting probes of the elephant (Yang *et al.* 2003*a*), hyrax and manatee were used to characterize conserved chromosomes and chromosome segments among the paenungulates and to compare each of these taxa with the outgroup species, the aardvark, using FISH. Hybridization experiments were carried out according to the methods described previously (Yang *et al.* 1997, 2003*a*).

### (c) Analysis

The patterns of chromosomal rearrangements detected between the paenungulates and the aardvark were scored according to the presence or the absence of discrete chromosomal homology characters. Each chromosomal rearrangement (character) was defined as either a fusion or a fission (character state; Dobigny *et al.* 2004) based on comparison with the aardvark, and additionally to data from human which are available indirectly through comparison with the aardvark and elephant (Frönicke *et al.* 2003; Yang *et al.* 2003*a*). Although human is not considered basal to afrotherians (Murphy *et al.* 2001; Waddell *et al.* 2001), the use of this species and the aardvark enables polarization of the character states for subsequent interpretation. In particular, among non-primate species, the aardvark has the lowest number of syntenic autosomal segments (in comparison to human) and also retains all of the proposed ancestral syntenies, indicating that the aardvark karyotype largely resembles that of the eutherian ancestor (Yang *et al.* 2003*a*; Robinson *et al.* 2004).

All rearrangements were characterized against aardvark chromosomes and this nomenclature was maintained across all comparisons to avoid scoring a particular character multiple times. Characters supporting the monophyly of retrieved clades, as well as those autapomorphic for individual lineages, were subsequently used to construct a phylogenetic tree (using maximum parsimony in PAUP^*^ v. 4.0b10; Swofford 2002) to which the characters were mapped.

## 3. Results

### (a) Flow sorting and assignment of paenungulate chromosomes

Flow sorting of the hyrax yielded usable paints for 25 out of the 27 hyrax chromosomes (2*n*=54), with chromosomes 26 and the X not identified among the sorted peaks. All 23 autosomes and the X and Y chromosomes (2*n*=48) of the manatee were identified among 23 separate peaks during flow sorting. Details and figures pertaining to the flow sorting of hyrax and manatee chromosomes are available in the electronic supplementary material, text S2, figure S3.

### (b) Cross-species chromosome painting

**Figure 1:**
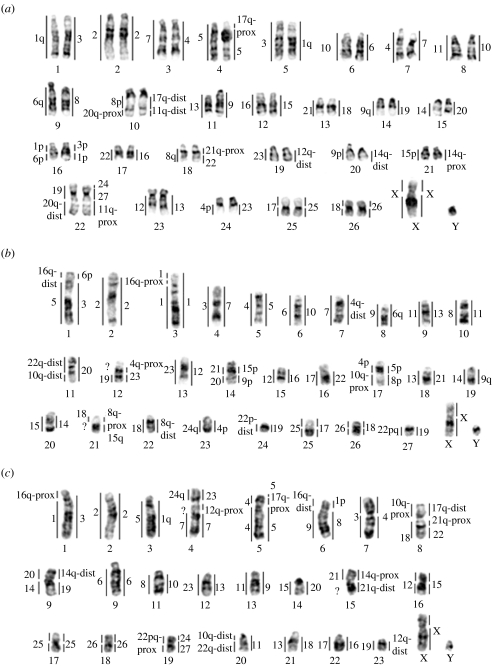
Summary of the cross-species painting results within Paenungulata mapped to G-banded metaphase chromosomes. (*a*) Chromosomes of the hyrax *P. capensis* (PCA), with regions homologous to the manatee (TMA, left) and elephant (LAF, right). (*b*) Chromosomes of the African elephant, *L. africana* with regions homologous to the manatee (right) and hyrax (left). (*c*) Chromosomes of the Florida manatee *T. m. latirostris* with regions homologous to the elephant (right) and hyrax (left). Question mark indicates chromosomal regions not resolved by cross-species painting.

**Figure 2:**
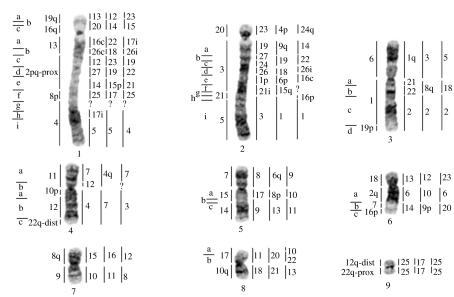
G-banded autosomes of the aardvark, *O. afer* (2*n*=20), with regions of homology (right of aardvark chromosomes) delimited by FISH to the elephant, manatee and hyrax (from left to right). Syntenies that were unclear on direct comparisons but confirmed through multispecies comparisons are marked by ‘c’ and those areas inferred from multispecies comparisons by ‘i’. Question mark indicates chromosomal regions not resolved by cross-species painting. Correspondence with human chromosomes is shown to the left of the aardvark chromosomes and is taken from Yang *et al.* (2003*a*). Positions of breakpoints are indicated to the left of human and each subdivided region is marked (a–i).

To characterize regions of chromosomal repatterning among the paenungulates, each set of chromosome probes from the three taxa was hybridized to each other reciprocally resulting in 29–33 synteny-conserved autosomal segments (figure 1). Hybridizations of painting probes of the elephant, manatee and hyrax to the aardvark delineated 36, 32 and 33 homologous segments, respectively. Homologous segments were mapped to G-banded chromosomes of the aardvark (figure 2) with homologies previously obtained from human probes also shown (Yang *et al.* 2003*a*). Details and additional figures pertaining to the painting trials are available in the electronic supplementary material, text S4, figure S5.

## 4. Discussion

### (a) Paenungulate-specific syntenies

**Figure 3:**
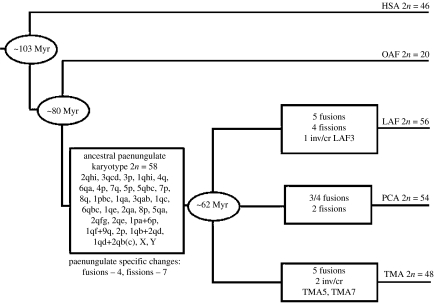
Phylogenetic tree based on chromosomal changes characterized in Paenungulata (elephant, LAF; manatee, TMA and hyrax, PCA) mapped relative to the outgroup taxon, the aardvark (OAF) (see figure 2 and text for details). Chromosome pairs deemed to have been present in the ancestral paenungulate (2*n*=58) are presented. Divergence estimates are taken from Springer *et al.* (2003). Inv, inversion; cr, centromere repositioning.

Comparative analysis of the distribution of chromosomal rearrangements between the paenungulates and the two outgroup taxa (aardvark and human) enabled the construction of a putative APK (figure 3). This hypothesized karyotype (2*n*=58) represents a paenungulate ancestor just prior to the divergence of Proboscidea, Hyracoidea and Sirenia, and allows for inferences on chromosomal evolution within this group relative to non-paenungulate afrotherian taxa.

Paenungulate-specific synapomorphies were identified and their specificity to this group was checked by comparisons with aardvark and golden mole (*Chrysochloris asiaticus*) and elephant shrew (*Elephantulus rupestris*) (Robinson *et al.* 2004). Confirmation of these syntenies awaits examination of the final member of Afrotheria, Tenrecomorpha, by comparative chromosome painting, and with missing data from elephant shrew–aardvark comparisons. A minimum estimate of 10 chromosomal changes (six fissions and four fusions) specific to Paenungulata was identified. The syntenic associations (figure 3) include OAF1pa+6p (HSA18/19q), OAF1qf+9q (HSA8p/22q), OAF1qd+2qb(c) (HSA2pqprox/3) and OAF1qb+2qd (HSA3q/13) and the fissions OAF1pa/1pb, OAF1qa/1qb, OAF 2qd/2qe, OAF2qe/2qf, OAF3qb/3qc and OAF5qa/5qb all of which were verified against data from other mammalian taxa (Froenicke 2005). These overlap with three of the eight previously reported (Yang *et al.* 2003*a*) elephant-specific segmental associations (HSA3/6, 18/19, 4/15, 2/16/7, 2/11, 4/16/19, 8/22 and 6/13/3). Further, segmental combinations that are found to be conserved across the four mammalian supraordinal groupings (HSA3/21, 7q/16, 12/22a, 14/15 and 16q/19q) were present in all three paenungulate taxa. The segmental associations of HSA3/5/21 and 1/19p were reported by Robinson *et al.* (2004) as afrotherian-specific syntenies (with a loss of chromosome 5 in the elephant for HSA3/5/21). Although HSA1/19p was evident in all three paenungulates in this study, recent results show this association to be present in the xenarthran *Tamandua tetradactyla* (Svartman *et al.* 2006; Yang *et al.* 2006). The status of this association as afrotherian specific (Frönicke *et al.* 2003; Robinson *et al.* 2004), or the alternative suggestion of an ancestral synteny (Yang *et al.* 2003*a*, 2006), is dependent on further investigation into whether the segments involved are indeed homologous. A fission within the HSA21 portion of the HSA3/5/21 synteny was found in the elephant by Frönicke *et al.* (2003) modifying this syntenic association to HSA3/21+HSA21/5 (OAF2qhi+OAF2qfg). This rearrangement was also present in both the elephant and the manatee in this study. The hyrax displays an additional fission (autapomorphic) within HSA21/5, but data corresponding to a part of the HSA3/21 (LAF1p/21) region are missing. The HSA3/21+ HSA21/5 fission present in the elephant and the manatee (and inferred in the hyrax) probably represents a change that occurred in the APK and is an additional synapomorphy for Paenungulata. It is important to note that these are inferred through hybridizations between human and aardvark, and the exact (positional) homologies require confirmation.

Examination of the chromosome painting data revealed five, five and three associations specific to *L. africana*, *T. m. latirostris* and *P. capensis*, respectively; however, no synapomorphies uniting any two paenungulate taxa were evident (see electronic supplementary material for additional information on paenungulate autapomorphies, text S6).

### (b) Rates of chromosomal evolution

Characterization of the number of unique changes within each paenungulate lineage facilitates the approximation of taxon-specific rates of evolution within Paenungulata (Waddell *et al.* 1999; Springer *et al.* 2003). Although estimates of evolutionary rate among taxa are dependent on several factors (e.g. generation time, population size), calculation of the number of changes observed over a defined period of time enables a comparison of the tempo of chromosomal evolution among different lineages (Dobigny *et al.* 2005*a*). Using this approach, a comparison of these rates indicates an elevated rate for the elephant (0.16 changes per Myr) in comparison with the hyrax (0.09 changes per Myr) and the manatee (0.11 changes per Myr) which agree more closely with the ‘default rate’ of mammalian chromosomal evolution estimated at 1 change/10 Myr (O'Brien & Stanyon 1999; Weinberg 2004). A more recent calculation sets this value at 1.9 changes/10 Myr (Froenicke 2005). However, estimates of chromosomal rates deduced from zoo-FISH experiments vary considerably within Eutheria. Elevated rates (5.8–33.3 changes per Myr; Dobigny *et al.* 2005*b*) have been observed within, e.g. Carnivora (Nash *et al.* 2001), Perissodactyla (Yang *et al.* 2003*b*), primates (Müller *et al.* 2003), Cervidae (Yang *et al.* 1997) and Muridae (Volobouev *et al.* 2002). In contrast, much reduced rates of evolution have been described from Rhinocerotidae (1 change over 17 Myr; Trifonov *et al.* 2003), Chrysochloridae (0.07 changes per Myr; Gilbert *et al.* 2006) and Xenarthra (0.2–0.4 changes per Myr; Dobigny *et al.* 2005*b*) to which rates estimated within Paenungulata are similar. Consequently, the chromosomal rates of evolution within this clade are best described as slow to moderate in comparison with other mammalian groups.

A comparison of homologous chromosomes and chromosomal segments among paenungulates indicates that the majority of whole chromosomes or chromosome arms have also maintained good G-band homology (figure S7 in electronic supplementary material). The majority of banding differences, based on the level of resolution here, appear to be characterized by minor rearrangements. Although G-banding is an indirect assessment of homology, and differences have been shown not necessarily to reflect underlying gene order evolution (Raudsepp & Chowdhary 1999), the degree of homology that appears to be maintained at the intrachromosomal level across all three taxa may be considered further evidence of a low rate of chromosomal evolution within Paenungulata.

A minimum estimate of the rate of chromosomal evolution in the ancestral paenungulate, i.e. prior to the divergence of Hyracoidea, Proboscidea and Sirenia, can be calculated using the minimum number of changes uniquely present in Paenungulata (11) and the estimated 18 Myr time period separating the divergence of the ancestral paenungulate from non-paenungulate afrotherians (approx. 80 Myr ago) and the radiation of the paenungulates (Springer *et al.* 2003). A rate of 0.61 changes per Myr is obtained which, although not as high as estimates for some other placental mammals, is approximately three to four times the fastest rate seen within paenungulates.

### (c) Chromosomal evolution within Paenungulata

Although FISH was performed on a single representative of each paenungulate order, the availability of cytogenetic data for other extant paenungulates enables an approximate intraordinal assessment of chromosomal evolution. In addition to *Loxodonta*, Proboscidea includes the genus *Elephas*, represented by the Asian elephant *Elephas maximus*. The G-banded karyotype (2*n*=56) of *E. maximus* is very similar to that of *L. africana*, with differences limited to heterochromatic and minor intrachromosomal variation (Houck *et al.* 2001), suggesting that almost no large chromosomal changes have occurred since their divergence approximately 6 Myr ago (Vignaud *et al.* 2002).

Within Hyracoidea, G-banded karyotypes exist for *Heterohyrax brucei* (2*n*=54) and *Dendrohyrax arboreus* (2*n*=54; Prinsloo & Robinson 1991), which show good correspondence to the karyotype of *P. capensis*. In particular, the chromosomes of *P. capensis* and *H. brucei* show a high degree of similarity (Prinsloo 1993). In contrast, *D. arboreus* displays differences most apparent in the distribution of heterochromatin particularly with respect to prominent heterochromatic short arms and terminal blocks of heterochromatin (Prinsloo & Robinson 1991). Based on fossil finds, a basal position for *Heterohyrax* with *Dendrohyrax* as the more derived genus has been suggested (McKenna & Bell 1997, p. 491) with a Middle–Late Miocene origin (approx. 10–14 Myr ago) for the modern family Procaviidae (Rasmussen *et al.* 1996). Molecular estimates for divergences within Procaviidae are similar to these fossil estimates (12.5–13.6 Myr ago for *Procavia* and *Dendrohyrax*; Springer 1997). The degree of karyotype conservation retained by *H. brucei* and *P. capensis* since their divergence is consistent with a reduced rate of chromosomal evolution within Hyracoidea as indicated by the chromosome painting data, although this may be an underestimate as suggested by the intrachromosomal differences detected between the Froenicke (2006) karyotype and that presented in this study (table S1 in electronic supplementary material). A Middle–Late Miocene origin provides ample opportunity for *Dendrohyra*-specific changes to have occurred. That these changes may be associated with the divergence event (e.g. Wichman *et al.* 1991) is also a consideration since although satellite sequences can remain dormant for extended periods, they are also capable of dynamic evolutionary changes (Ugarković & Plohl 2002).

In contrast to proboscideans and hyracoids, the sirenians exhibit the greatest variation in chromosome number (2*n*=48–56) among paenungulates for which cytogenetic data are available. These include the Florida manatee, 2*n*=48 (Gray *et al.* 2002), the Amazonian manatee, *Trichechus inunguis*, 2*n*=56 (Assis *et al.* 1988) and the dugong, *Dugong dugon*, 2*n*=50 (Short 1984). Both G- and C-banding data for the Amazonian manatee are available for comparison with the Florida manatee. Although C-banding patterns are restricted to the centromeres of both species (Assis *et al.* 1988; Gray *et al.* 2002), differences between G-banding patterns are more extensive. In particular, the difference in chromosome number indicates that at least four chromosomal changes separate these two taxa. Phylogenetic analyses of mtDNA sequence data suggest that *T. inunguis* and *T. manatus* diverged approximately 1–4 Myr ago (Catanhede *et al.* 2005; Vianna *et al.* 2005) in agreement with fossil evidence (Domning 1982). This suggests a far greater degree of chromosomal change (1–4 changes per Myr) over the last 4 Myr in Sirenia in comparison with that seen in either of the other paenungulate lineages.

Using the estimated rates of genomic change calculated above, a pattern of paenungulate chromosomal evolution spanning approximately 80 Myr can be described. This entails a reduction of the rate within Paenungulata relative to the ancestral paenungulate and this is maintained across all three lineages with the exception of increased repatterning evident in the last 4 Myr of sirenian evolution. Consequently, where it is assumed that the radiation of Paenungulata involves two distinct dichotomous events rather than a simultaneous ‘trivergence’, the absence of a chromosomal synapomorphy uniting two paenungulates (at the level of resolution characterized in this study) may be due to a reduced rate of chromosomal change relative to the length of time separating successive divergence events. Based on the divergence estimate of approximately 62 Myr ago (Springer *et al.* 2003) and the earliest documented appearance (55–58 Myr ago) of the first primitive paenungulate (the proboscidean *Phosphatherium escuilliei*; Gheerbrant *et al.* 1996), a period of 4–7 Myr is estimated for the radiation of Paenungulata into the three extant lineages. Taking into account the divergence between *T. manatus* and *T. inunguis* over a similar length of time (approx. 4 Myr), the absence of synapomorphies indicates that the divergence of Paenungulata is not associated with significant chromosomal repatterning, and consequently does not seem to be coincident with a period of increased rate of chromosomal change as suggested for the recent trichechid divergence and as described for other mammalian taxa (e.g. Britton-Davidian *et al.* 2000; Nash *et al.* 2001; Volobouev *et al.* 2002; Navarro & Barton 2003; Dobigny *et al.* 2005*a*).

In conclusion, this study represents the first investigation into the chromosomal evolution among the three paenungulate lineages and provides confirmation of the monophyly of Paenungulata using cytogenetic characters. Although no synapomorphic changes defining intrapaenungulate associations were evident, this may simply reflect the limitations of zoo-FISH, and continued investigation of the paenungulate polytomy is warranted. This will benefit greatly from the sequencing of the elephant genome (Roca & O'Brien 2005), which will in turn help to place paenungulate genome organization in the proper context within Mammalia.

## Supplementary Material

Pardini *et al.* electronic appendix Chromosome painting among Proboscidea, Hyracoidea and Sirenia: support for Paenungulata (Afrotheria, Mammalia) but not Tethytheria

## References

[bib1] Amrine H.M, Springer M.S (1999). Maximum-likelihood analysis of the Tethythere hypothesis based on a multigene data set and a comparison of different models of sequence evolution. J. Mamm. Evol.

[bib2] Amrine-Madsen H, Koepfli K.P, Wayne R.K, Springer M.S (2003). A new phylogenetic marker, apolipoprotein B, provides compelling evidence for eutherian relationships. Mol. Phylogenet. Evol.

[bib3] Assis M.F.L, Best R.C, Barros R.M.S, Yonenaga-Yassuda Y (1988). Cytogenetic study of *Trichechus inunguis* (Amazonian manatee). Rev. Bras. Genet.

[bib4] Britton-Davidian J, Catalan J, Ramalhinho M.G, Ganem G, Auffray J.-C, Capela R, Biscoito M, Searle J.B, Mathias M.L (2000). Rapid chromosomal evolution in island mice. Nature.

[bib5] Catanhede A.M, Da Silva V.M.F, Farias I.P, Hrbek T, Lazzarini S.M, Alves-Gomes J (2005). Phylogeography and population genetics of the endangered Amazonian manatee, *Trichechus inunguis* Natterer, 1883 (Mammalia, Sirenia). Mol. Ecol.

[bib8] Dobigny G, Ducroz J.-F, Robinson T.J, Volobouev V (2004). Cytogenetics and cladistics. Syst. Biol.

[bib6] Dobigny G, Aniskin V, Granjon L, Cornette R, Volobouev V (2005a). Recent radiation in West African *Taterillus* (Rodentia, Gerbillinae): the concerted role of chromosome and climatic changes. Heredity.

[bib7] Dobigny G, Yang F, O'Brien P.C.M, Volobouev V, Kovács A, Pieczarka J.C, Ferguson-Smith M.A, Robinson T.J (2005b). Low rate of genomic repatterning in Xenarthra inferred from chromosome painting data. Chromosome Res.

[bib9] Domning D.P (1982). Evolution of manatees. A speculative history. J. Paleontol.

[bib10] Ferguson-Smith M.A, Yang F, O'Brien P.C.M (1998). Comparative mapping using chromosome sorting and painting. ILAR J.

[bib11] Fischer M.S, Tassy P, McKenna M.C (1993). The interrelation between Proboscidea, Sirenia, Hyracoidea, and Mesaxonia: the morphological evidence. Mammal phylogeny: placentals.

[bib12] Froenicke L (2005). Origins of primate chromosomes—as delineated by Zoo-FISH and alignments of human and mouse draft genome sequences. Cytogenet. Genome Res.

[bib13] Froenicke L, O'Brien S.J, Menninger J.C, Nash W.G (2006). A G-banded karyotype of the rock hyrax (*Procavia capensis*). Atlas of mammalian chromosomes.

[bib14] Frönicke L, Wienberg J, Stone G, Adams L, Stanyon R (2003). Towards the delineation of the ancestral eutherian genome organization: comparative genome maps of human and the African elephant (*Loxodonta africana*) generated by chromosome painting. Proc. R. Soc. B.

[bib14a] Gheerbrant E, Sudre J, Cappetta H (1996). A palaeocene proboscidean from morocco. Nature.

[bib15] Gheerbrant E, Domning D.P, Tassy P, Rose K.D (2005). Paenungulata (Sirenia, Proboscidea, Hyracoidea, and relatives). The rise of placental mammals.

[bib16] Gilbert C, O'Brien P.C, Bronner G, Yang F, Hassanin A, Ferguson-Smith M.A, Robinson T.J (2006). Chromosome painting and molecular dating indicate a low rate of chromosomal evolution in golden moles (Mammalia, Chrysochloridae). Chromosome Res.

[bib17] Gray B.A, Zori R.T, McGuire J.A, Bonde R.K (2002). A first generation cytogenetic ideogram for the Florida manatee (*Trichechus manatus latirostris*) based on multiple chromosome banding techniques. Hereditas.

[bib18] Houck M.L, Kumamoto A.T, Gallagher D.S, Benirschke K (2001). Comparative cytogenetics of the African elephant (*Loxodonta africana*) and Asiatic elephant (*Elephas maximus*). Cytogenet. Cell Genet.

[bib19] McKenna M, Luckett W.P, Szalay F.S (1975). Toward a phylogenetic classification of the Mammalia. Phylogeny of the primates.

[bib20] McKenna M.C, Bell S.K (1997). Classification of mammals above the species level.

[bib21] Müller S, Hollatz M, Wienberg J (2003). Chromosomal phylogeny and evolution of gibbons (Hylobatidae). Hum. Genet.

[bib22] Murata Y, Nikaido M, Sasaki T, Cao Y, Fukumoto Y, Hasegawa M, Okada N (2003). Afrotherian phylogeny as inferred from complete mitochondrial genomes. Mol. Phylogenet. Evol.

[bib23] Murphy W.J (2001). Resolution of the early placental mammal radiation using Bayesian phylogenetics. Science.

[bib24] Nash W.G, Menninger J.C, Wienberg J, Padilla-Nash H.M, O'Brien S.J (2001). The pattern of phylogenomic evolution of the Canidae. Cytogenet. Cell Genet.

[bib25] Navarro A, Barton N.H (2003). Chromosomal speciation and molecular divergence—accelerated evolution in rearranged chromosomes. Science.

[bib26] Nikaido M, Cao Y, Harada M, Okada N, Hasegawa M (2003). Mitochondrial phylogeny of hedgehogs and monophyly of Eulipotyphla. Mol. Phylogenet. Evol.

[bib27] Nishihara H, Satta Y, Nikaido M, Thewissen J.G.M, Stanhope M.J, Okada N (2005). A retroposon analysis of afrotherian phylogeny. Mol. Biol. Evol.

[bib28] O'Brien S.J, Stanyon R (1999). Ancestral primate viewed. Nature.

[bib29] Prinsloo, P. 1993 Molecular and chromosomal phylogeny of the Hyracoidea. Ph.D. thesis, Department of Zoology, University of Pretoria, p. 130.

[bib30] Prinsloo P, Robinson T.J (1991). Comparative cytogenetics of the Hyracoidea: chromosomes of two *Hyrax* species from South Africa. Z. Säugetierkunde.

[bib31] Rasmussen T.D, Pickford M, Mein P, Senut B, Conroy G.C (1996). Earliest known Procaviid hyracoid from the late Miocene of Namibia. J. Mammal.

[bib32] Raudsepp T, Chowdhary B.P (1999). Construction of chromosome-specific paints for meta- and submetacentric autosomes and the sex chromosomes in the horse and their use to detect homologous chromosomal segments in the donkey. Chromosome Res.

[bib33] Robinson T.J, Seiffert E.R (2004). Afrotherian origins and interrelationships: new views and future prospects. Curr. Top. Dev. Biol.

[bib34] Robinson T.J, Fu B, Ferguson-Smith M.A, Yang F (2004). Cross-species chromosome painting in the golden mole and elephant–shrew: support for the mammalian clades of Afrotheria and Afroinsectiphillia but not Afroinsectivora. Proc. R. Soc. B.

[bib35] Roca A.L, O'Brien S.J (2005). Genomic inferences from Afrotheria and the evolution of elephants. Curr. Opin. Genet. Dev.

[bib36] Rokas A, Holland P.W.H (2000). Rare genomic changes as a tool for phylogenetics. Trends Ecol. Evol.

[bib37] Short R.V, Garlick D.G, Korner P.I (1984). Hopping mad. Frontiers in physiological research.

[bib38] Simpson G.G (1945). The principles of classification and a classification of mammals. Bull. Am. Mus. Nat. Hist.

[bib39] Springer M.S (1997). Molecular clocks and the timing of the placental and marsupial radiations in relation to the Cretaceous/Tertiary boundary. J. Mamm. Evol.

[bib40] Springer M.S, Murphy W.J, Eizirik E, O'Brien S.J (2003). Placental mammal diversification and the Cretaceous–Tertiary boundary. Proc. Natl Acad. Sci. USA.

[bib41] Svartman M, Stone G, Stanyon R (2006). The ancestral Eutherian karyotype is present in Xenarthra. PLoS Genet.

[bib42] Swofford, D. L. 2002 *PAUP*^*^: *phylogenetic analysis using parsimony* (^*^*and other methods*). Sunderland, MA: Sinauer.

[bib43] Telenius H, Carter N.P, Bebb C.E, Nordenskjöld M, Ponder B.A.J, Tunnacliffe A (1992). Degenerate oligonucleotide-primed PCR: general amplification of target DNA by a single degenerate primer. Genomics.

[bib44] Trifonov V, Yang F, Ferguson-Smith M.A, Robinson T.J (2003). Cross-species chromosome painting in the Perissodactyla: delimitation of homologous regions in Burchell's zebra (*Equus burchellii*) and the white (*Ceratotherium simum*) and black rhinoceros (*Diceros bicornis*). Cytogenet. Genome Res.

[bib45] Ugarković Ð, Plohl M (2002). Variation in satellite DNA profiles—causes and effects. EMBO J.

[bib46] Vianna J.A (2005). Phylogeography, phylogeny and hybridization in trichechid sirenians: implications for manatee conservation. Mol. Ecol.

[bib47] Vignaud P (2002). Geology and palaeontology of the Upper Miocene Toros-Menalla hominid locality, Chad. Nature.

[bib48] Volobouev V.T, Aniskin V.M, Lecompte E, Ducroz J.-F (2002). Patterns of karyotype evolution in complexes of sibling species within three genera of African murid rodents inferred from the comparison of cytogenetic and molecular data. Cytogenet. Genome Res.

[bib49] Waddell P.J, Cao Y, Hasegawa M, Mindell D.P (1999). Assessing the Cretaceous superordinal divergence times within birds and placental mammals by using whole mitochondrial protein sequences and an extended statistical framework. Syst. Biol.

[bib50] Waddell P.J, Kishino H, Ota R (2001). A phylogenetic foundation for comparative mammalian genomics. Genome Inform.

[bib51] Weinberg J (2004). The evolution of eutherian chromosomes. Curr. Opin. Genet. Dev.

[bib52] Wichman H.A, Payne C.T, Ryder O.A, Hamilton M.J, Maltbie M, Baker R.J (1991). Genomic distribution of heterochromatic sequences in equids: implications to rapid chromosomal evolution. J. Hered.

[bib53] Yang F, O'Brien P.C.M, Wienberg J, Neitzel H, Lin C.C, Ferguson-Smith M.A (1997). Chromosomal evolution of the Chinese muntjac (*Muntiacus reevesi*). Chromosoma.

[bib54] Yang F (2003a). Reciprocal chromosome painting among human, aardvark, and elephant (superorder Afrotheria) reveals the likely eutherian ancestral karyotype. Proc. Natl Acad. Sci. USA.

[bib55] Yang F, Fu B, O'Brien P.C.M, Robinson T.J, Ryder O.A, Ferguson-Smith M.A (2003b). Karyotypic relationships of horses and zebras: results of cross-species chromosome painting. Cytogenet. Genome Res.

[bib56] Yang F (2006). Comparative genome maps of the pangolin, hedgehog, sloth, anteater and human revealed by cross-species chromosome painting: further insight into the ancestral karyotype and genome evolution of eutherian mammals. Chromosome Res.

