# Supplementary material for: Chromosome painting among Proboscidea, Hyracoidea and Sirenia: support for Paenungulata (Afrotheria, Mammalia) but not Tethytheria
Source: Proc Biol Sci. 2007 Mar 20;274(1615):1333–40. doi: 10.1098/rspb.2007.0088 (PMC1914331; doi:10.1098/rspb.2007.0088)
Supplement: Pardini et al. electronic appendix — Chromosome painting among Proboscidea, Hyracoidea and Sirenia: support for Paenungulata (Afrotheria, Mammalia) but not Tethytheria [file rspb20070088s21.doc]

ELECTRONIC APPENDIX

**CHROMOSOME PAINTING AMONG PROBOSCIDEA, HYRACOIDEA AND SIRENIA: SUPPORT FOR PAENUNGULATA (AFROTHERIA, MAMMALIA) BUT NOT TETHYTHERIA**

**by**

**A.T. Pardini, P. C. M. O’Brien, B. Fu, R.K. Bonde, F.F.B. Elder, M.A. Ferguson- Smith, F. Yang, T.J. Robinson**

**Supporting table S1: Suspected positional differences between the *Procavia capensis* G-banded karyotypes published by Froenicke (2006) and the specimen analysed in the present study.**

| This study | Froenicke 2006 |
| --- | --- |
| 1 | 3 |
| 2 | 1 |
| 3 | 2 |
| 4 | 5 |
| 5 | 4 |
| 6 | 6 |
| 7 | 9 |
| 8 | 10 |
| 9 | 7 |
| 10 | 8 |
| 11 | 11 |
| 12 | 12 |
| 13 | 15 or 13? |
| 14 | 14 |
| 15 | 19? |
| 16 | 16 |
| 17 | 17 |
| 18 | 13? |
| 19 | 18 |
| 20 | 20 |
| 21 | 21 |
| 22 | 22 |
| 23 | 23 |
| 24 | 24 |
| 25 | 25? |
| 26 | 26? |

**Supporting text S2: Flow sorting and assignment of paenungulate chromosomes.**

*(i) Hyrax*

Of the 24 painting probes generated for the rock hyrax (supporting figure S3*a*), 19 hybridised to a single chromosome (numbers 1, 2, 4, 6, 7, 9, 11, 12, 13, 14, 15, 17, 18, 20, 21, 22, 23, 25, Y). The two homologues of chromosomes 15, 16 and 25 were each sorted into a different peak. Pure sorts for chromosomes 15 and 25 were also generated and peaks containing these chromosomes were consequently resolvable. Both homologues of chromosome 16 were each isolated with a different chromosome (15 and 24 respectively) allowing for their use as chromosome-specific paints by two-colour FISH. Two flow-peaks, each containing chromosomes 3+5 and 8+10, could not be resolved further. Chromosome 26 and the X were not identified in the peaks that were sorted.

*(ii) Manatee*

The 48 chromosomes of the manatee separated into 23 peaks (supporting figure S3*b*) of which 17 each contained a single chromosome (numbers 1, 5, 8-12, 14-18, 20, 20, 22-23, Y). Chromosome 20 was present in two separate peaks. The homologues of chromosomes 17 and 11 co-sorted with chromosomes 21 and 13 respectively. Although chromosome 19 exists with chromosome 14, a pure form of 14 was sorted which allows characterisation of chromosome 19 in interspecific hybridisations. Chromosomes contained in peaks 3+7, 2+4 and 6+X could not be resolved further.

Chromosomes that were not resolved individually were characterised in the subsequent zoo-FISH trials involving multi-species comparisons. For example, hyrax chromosome 26 was not identified during flow-sorting yet, the elephant and manatee probes that hybridise to PCA26 also hybridised to each other, confirming that these homologies exist among all three paenungulates.


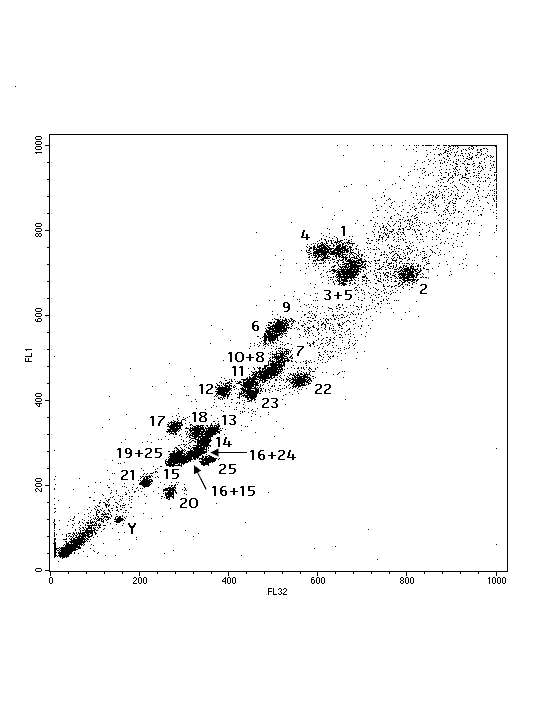


**Hoeschst fluorescence**

0 200 400 600 800 1000

0 200 400 600 800 1000

**Chromomycin fluorescence**

**a**


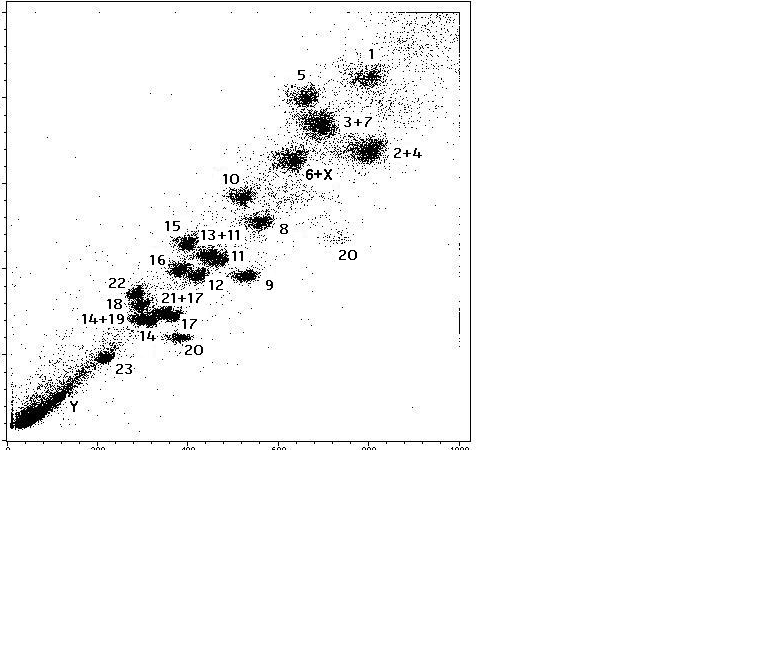


0 200 400 600 800 1000

**Chromomycin fluorescence**

**Hoeschst fluorescence**

0 200 400 600 800 1000

**b**

**Supporting figure S3:** Assignment of bivariate-sorted chromosomes using FISH to G-banded chromosomes of a) the hyrax, *P. capensis* (2n=54, XY), and b) the manatee, *T. m. latirostris* (2n=48, XY).

**Supporting text S4: Results of cross species chromosome painting experiments among hyrax, elephant, manatee and the outgroup species, the aardvark.**

*(i) Hyrax*

Hybridisation experiments of elephant and manatee painting probes to hyrax chromosomes are depicted in figure 1*a* and yielded 32 and 29 synteny-conserved autosomal segments, respectively. Seventeen manatee (2, 3, 5, 7, 10-19, 21-23) chromosomes and 22 elephant autosomes (2, 4-10, 12, 13, 15, 16, 18-27) and the X chromosome were conserved in their entirety, while the remaining six manatee (1, 4, 6, 8, 9, 20) and five elephant (1, 3, 11, 14, 17) probes produced two signals each. The manatee X chromosome was flow-sorted with chromosome 6. When this painting probe was applied to hyrax chromosomes, a signal was produced covering the X chromosome in addition to the autosomal segments. There are three regions present on hyrax chromosomes (10q-prox, 23p and 24p) for which no signal was detected with respect to hybridisations with either elephant or manatee probes and may represent the missing signals on TMA15q, TMA4q-prox, LAF12q-prox, LAF21q-dist and LAF20q-dist (described below).

*(ii) Elephant*

The G-banded chromosomes of the elephant with areas of homology to the hyrax and the manatee based on zoo-FISH experiments are shown in figure 1*b*. The hyrax and manatee painting probes identified 33 and 32 conserved homologous autosomal segments in the elephant, respectively. Twenty (2, 3, 5-9, 11-15, 17, 19-21, 23-26) and sixteen (1-3, 7, 10-14, 16-18, 20-23) autosomal syntenic groups that are conserved *in toto* were apparent between the hyrax and elephant, and the manatee and elephant, respectively. The remaining five hyrax chromosomes (1, 4, 10, 16, 18, 22) delineated 2-3 segments each while LAF12qprox, LAF21q-dist and LAF20q-dist did not show any hybridisation signals when painted with hyrax probes. Seven (4, 5, 6, 8, 9, 15, 19) manatee chromosomes produced 2-3 signals each when painted to the elephant. LAF20q-dist did not appear to hybridise with any manatee probe. The painting probe derived from manatee flow-peak 6+X hybridised to two autosomal segments in addition to the elephant X chromosome.

*(iii) Manatee*

Areas of homology between elephant and hyrax probes and manatee metaphase chromosomes are shown in figure 1*c*. Thirty-three segments of conserved synteny are evident between the elephant and manatee chromosomes, and 29 regions were found between the hyrax and manatee chromosomes. Elephant chromosomes 1, 5, 12, 14, 17 and 21 each produced signals on two different chromosomes in the sirenian, while the remaining 21 elephant chromosomes 2-4, 6-11, 13, 15, 16, 18-20 and 22-27 each painted to one manatee chromosome or chromosome arm. Hyrax chromosomes 10, 16 and 22 each produced signals on two different chromosomes in the sirenian, while the remaining 23 autosomes (1-9, 11-15, 17-21, 23-26) each painted to one manatee chromosome or chromosome arm. Only the pattern showing hybridisation of the elephant X chromosome to manatee is indicated as this chromosome was not identified during the flow-sorting of the hyrax chromosomes (see above). Hybridisation experiments with hyrax did not produce signals on regions TMA4q-prox and TMA15q.

*(iv) Aardvark*

Refer to figure 3 in main article.

Of the 36 different segments delineated by elephant probes, 18 elephant chromosomes each correspond to one segment (2-11, 15, 16, 18-20, 22-24) while the remaining nine chromosomes (1, 12-14, 17, 21, 25-27) each correspond to two different segments. Three (16c, 26c, 1c) of the 36 segments delineated by the elephant were not clear from direct hybridisations but were confirmed through multispecies comparisons. A further two signals (21i and 17i), expected from painting results with other taxa (hyrax and manatee), were not found but were inferred from results with other species.

Of the 32 homologous segments delineated by manatee probes to aardvark chromosomes, 14 manatee autosomes (1-3, 5, 7, 10, 11, 13, 14, 16, 20-23) each correspond to one segment while the remaining nine chromosomes (4, 6, 8, 9, 12, 15, 17-19) each hybridised to two segments. Hybridisations of hyrax probes to aardvark delineated 33 conserved segments of which 20 (1-9, 11-15, 17-21, 24) produced a single signal, five (10, 16, 23, 25, 26) produced two signals and a single chromosome (22) was found to hybridise to three different aardvark chromosomes. Three different segments (26i, 26i, 17i) were not obtained directly during hybridisation trials; chromosome 26, like the X chromosome, was not identified during flow-sorting of the hyrax while 17 was inferred through multispecies comparisons. A further signal which was not clear through direct hybridisations was confirmed through multispecies comparisons (16c).

All three paenungulate species did not hybridise to a small section on the q-arm of aardvark chromosome 1, and in addition, paints showed limited hybridisation to the syntenic section of OAF3p. We base this latter assignment on the Yang et al. (2003) data. A segment on OAF2q corresponding to the homologous regions of LAF21q-dist and TMA15q which did not yield signals during hybridisation trials with the hyrax, was also not detected during hyrax to aardvark hybridisations.


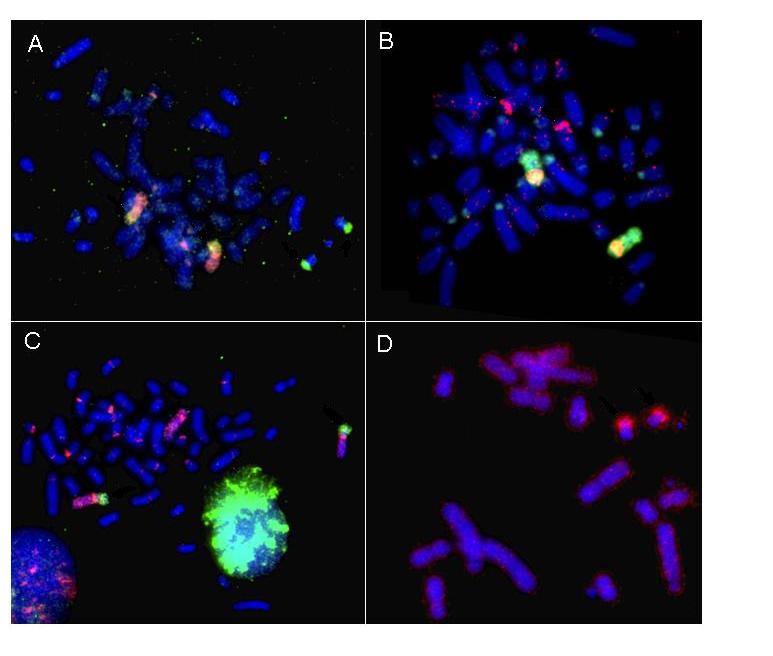


**Supporting figure S5:** Examples of cross-species chromosome painting using painting probes developed specifically for this investigation: (a) two-colour FISH on LAF metaphase chromosomes using avidin-CY3 (pink) labelled LAF17 and antidigoxigenin-FITC (green) labelled TMA8; (b) two-colour FISH on PCA chromosomes using antidigoxigenin-FITC (green) labelled PCA22 and avidin-CY3 (pink) labelled TMA20; (c) two-colour FISH on TMA metaphase chromosomes using avidin-CY3 (pink) labelled TMA3+4 (mixed peak) and antidigoxigenin-FITC (green) labelled LAF23; (d) OAF metaphase chromosomes painted with avidin-CY3 (pink) labelled TMA20. Note: where the two probes overlap, the colour appears yellow/orange; chromosomes are labelled with DAPI (blue).

**Supporting text S6: Paenungulate-specific syntenies**

Elephant-specific syntenies HSA3/6, 4/15, 2/16/7, 2/11 reported in Yang et al. (2003) were confirmed but not 6/13/3. The presence of HSA6 on LAF26 was ambiguous in the original study and was not found here. Further, HSA2/11 involves an unclear designation in the hyrax and may be excluded with the addition of new data. In agreement with Frönike et al. (2003) the association uniting OAF3qa+2qfg (HSA1/3/21) was found in the elephant. Syntenies found in the hyrax include OAF5qa+8pa (HSA15/17), OAF(2qbc+1qd)+8pb (HSA2pqprox/17) and OAF2qh+2qe (HSA3/21) and those specific to the manatee comprise OAF2p+4pb (HSA11/20), OAF2qe+5p (HSA3/7), OAF5qa+3qab (HSA1/15), OAF6qbc+2qa (HSA7/16p/3) and OAF1qe+2qfg (HSA2pqprox/21) as well as an inversion/centromere repositioning (Amor et al. 2004; Montefalcone et al. 1999; Ventura et al. 2004) on TMA5 and TMA7.

**
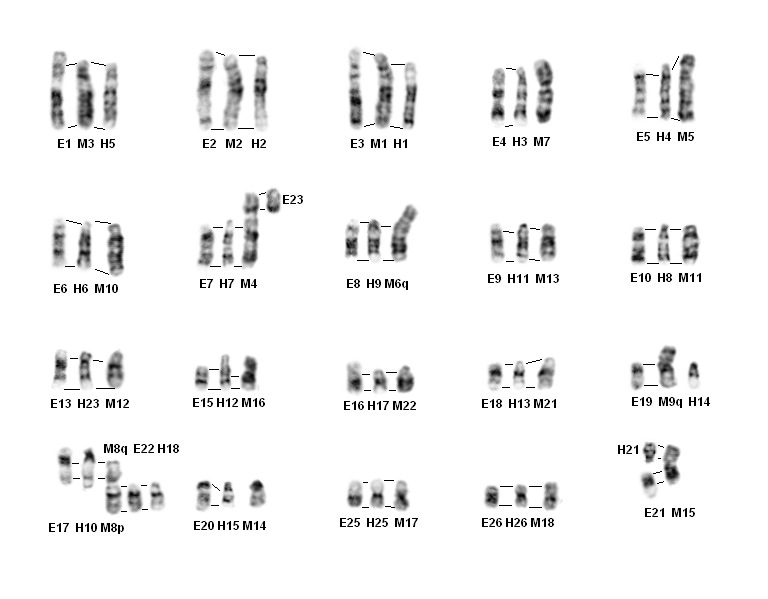
**

**Supporting figure S7:** Comparison of G-banded chromosomes of the elephant (E), hyrax (H) and manatee (M). Numbers correspond to chromosomes in the standard karyotypes of the respective species. Horizontal bars indicate homologies as assessed by FISH. Where no bars are indicated, the G-band similarity is not obvious although chromosomal homology has been defined by FISH.

**S8: Supplementary information references**

Amor, D. J., Bentley, K., Ryan, J., Perry, J., Wong, L., Slater, H. & Choo, K. H. A. 2004 Human centromere repositioning "in progress". *Proc. Natl. Acad. Sci. USA* **101**, 6542-6547. (doi:10.1073/pnas.0308637101)

Frönicke, L., Wienberg, J., Stone, G., Adams, L. & Stanyon, R. 2003 Towards the delineation of the ancestral eutherian genome organization: comparative genome maps of human and the African elephant (*Loxodonta africana*) generated by chromosome painting. *Proc. R. Soc. B* **270**, 1331-1340. (doi:10.1098/rspb.2003.2383)

Montefalcone, G., Tempesta, S., Rocchi, M. & Archidiacono, N. 1999 Centromere repositioning. *Genome Res.* **9**, 1184-1188. (doi:10.1101/gr.9.12.1184 )

Ventura, M., Weigl, S., Carbone, L., Cardone, M. F., Misceo, D., Teti, M., D'Addabbo, P., Wandall, A., Bjorck, E., De Jong, P. J., She, X., Eichler, E. E., Archidiacono, N. & Rocchi, M. 2004 Recurrent sites for new centromere seeding. *Genome Res.* **14**, 1696-1703. (doi:10.1101.gr.2608804)

Yang, F., Alkalaeva, E. Z., Perelman, P. L., Pardini, A. T., Harrison, W. R., O'Brien, P. C. M., Fu, B., Graphodatsky, A. S., Ferguson-Smith, M. A. & Robinson, T. J. 2003 Reciprocal chromosome painting among human, aardvark, and elephant (superorder Afrotheria) reveals the likely eutherian ancestral karyotype. *Proc. Natl. Acad. Sci. USA* **100**, 1062-1066. (doi:10.1073/pnas.0335540100)
